# Isolated Peroneus Longus Tear - Commonly Missed Diagnosis of Lateral Ankle Pain: A Case Report

**DOI:** 10.5704/MOJ.2007.011

**Published:** 2020-07

**Authors:** AF Abd-Rasid, MY Bajuri

**Affiliations:** Department of Orthopaedics and Traumatology, Universiti Kebangsaan Malaysia Medical, Kuala Lumpur, Malaysia

**Keywords:** persistent ankle pain, peroneus longus, functional disability

## Abstract

Peroneal tendon tear is a relatively common cause of lateral ankle pain but often missed due to mixed presentation or low index of suspicion. Left untreated, peroneal injuries can lead to persistent ankle pain, instability and ultimately substantial functional disabilities. An isolated peroneus longus tear is rare with the lowest incidence rate compared to isolated peroneus brevis tear and mixed tear of both peroneal tendon. This is a case report of a 49-year-old lady with a chronic left ankle pain who ultimately underwent surgery for an isolated peroneus longus tear.

## Introduction

The peroneal muscles are the pronators and evertors of the foot. They also act as a dynamic stabiliser of the ankle joint and are important in maintaining proprioception of the foot independent of the status of lateral ankle ligaments. A compromised peroneal function may lead to a sense of instability and eventually causing persistent pain and occasional swelling of the ankle. Although the true incidence of peroneal tendon tears is unknown, an estimated range is from 11% to 37% in cadaveric dissections and up to 30% in patients undergoing surgery for ankle instability^1, 2^. The aetiology of peroneal tendon tears is not fully understood, however, they are likely caused by a combination of mechanical and local anatomical factors, such as the anatomy of the peroneal groove, the presence of tendon subluxation or dislocation, the integrity of the superior peroneal retinaculum and the presence of ankle instability. Although controversial, some authors proposed points of critical avascular zones throughout the peroneus brevis and longus tendon which may contribute to the tendinopathy^2, 3^.

Patients with peroneal tendinitis or peroneal tendon tear will complain of pain over the posterior aspect of the lateral malleolus at the area of the peroneal tendons which is worsen by passive inversion of the hind foot and plantarflexion of the ankle joint or even by active resisted hind foot eversion. On physical examination, there is tenderness along the course of the peroneal tendons. Patient will also occasionally present with swelling and warmness over the lateral malleolus especially during acute phase of the tendinitis. The alignment of the foot should be inspected as a cavovarus foot is associated with increase rate of peroneal tendon disorders^2-4^.

Weight-bearing radiographs of the foot and ankle may reveal osseous lesions that can be associated with peroneal tendinopathy otherwise, radiographs are more useful in excluding other causes of lateral ankle foot pain such as fractures and presence of accessory bones^2^. The hindfoot alignment radiograph can help to identify cavovarus foot which as mentioned earlier is a predisposing cause for peroneal tendinopathy. Ultrasonography can be helpful in evaluation of peroneal tendon as it can detect accumulation of peritendinous fluid and thickening of the tendon^3^. However, it is highly operator-dependent and surgeons prefer to use magnetic resonance imaging (MRI) for diagnostic and pre-operative planning purposes. Magnetic resonance imaging remains the gold standard for evaluating tendon disorders^2, 4^.

## Case Report

A 49-year-old female with underlying hypertension and well-controlled diabetes mellitus for the past six years, came to us with a history of pain in the left foot and ankle since two years ago, mainly over the heel and around the lateral malleolus (following standing or walking) that has gotten worse in the past months and developed occasional swelling and warmth. Upon examination, there was no significant finding other than tenderness along lateral aspect of lateral malleolus. There was no swelling or warmth, and walking was painless.

She was diagnosed with plantar fasciitis. Following three months of conservative management, heel pain abated, but lateral malleolar pain remained become more painful, however, no swelling or redness was observed.

Ultrasonography showed tendinopathy of the tendons of peroneus longus and brevis. Following peroneal stretching and strengthening physiotherapy for six months, the patient reported no improvement, and was advised to undergo surgery despite pre-operative MRI revealed that complete tear was absent for both peroneal tendons.

A 10cm hockey-stick/curve incision posterior to lateral malleolus directly over the tendon. Sural nerve was identified and protected. Both the superior and the inferior peroneal retinaculum were intact. Subluxation of the long peroneal tendon was observed upon passive dorsiflexion and inversion.

The intra-operative findings revealed that the peroneus brevis tendon was intact (Fig. 2). However, there was a longitudinal tear of the peroneus longus tendon (Fig. 2) with surrounding inflammation and degenerative changes.

**Fig. 1:**
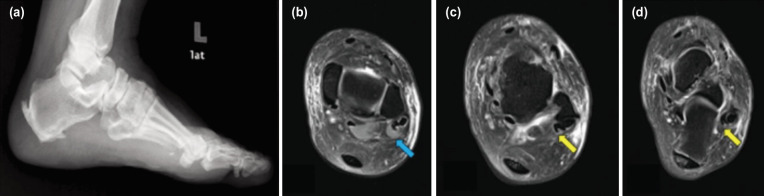
(a) Presence of calcaneal spur on lateral view of left foot and ankle radiograph. (b) The blue arrow showing ankle joint’s MRI of the peritendinous fluid within the peroneal tendon sheath. (c, d) The yellow arrows showing ankle joint’s MRI of longitudinal tear of either peroneal tendons.

**Fig. 2:**
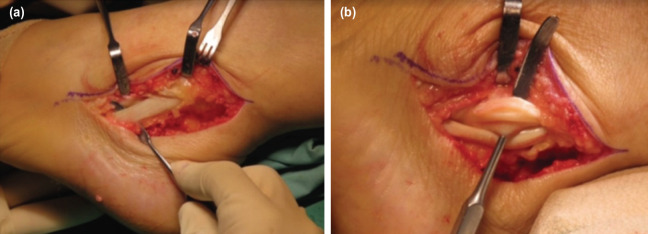
(a) Intra-operative finding showing intact peroneal brevis tendon. (b) Intra-operative finding showing longitudinal tear of the peroneal longus tendon.

**Fig. 3:**
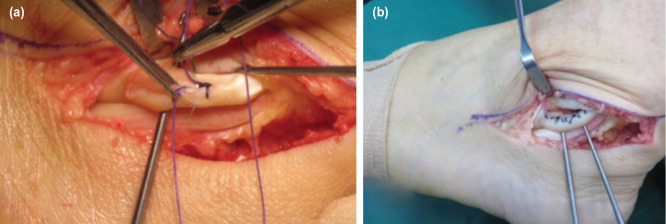
(a) Showing tubularization of the peroneal longus tendon. (b) The degenerative tissues were debrided prior to the procedure.

Next, debridement of the peroneus longus and primary repair with tubularization of the tendons was done (Fig. 3). A 4.0 braided absorbable suture was used for the running locking suturing. This suture is chosen as it causes less irritation to the surrounding tissue and less inflammation.

Post-operatively, patient was immobilised in a fibre-glass bootslab for six weeks before the physical therapy program. The post-operative protocol is designed to emphasise early range of motion. The patient was advised upon non-weight bearing ambulation for two weeks in a splint and then was allowed to be weight bearing as tolerated in an air walker for the following four weeks. At six weeks post-operation, she was advised to remove the air walker and start to walk with full weight bearing. This physical therapy regime emphasised on regaining strength as well as proprioception property of the peroneal muscles. The patient was walking pain-free compared to her pre-operative condition, as she was experiencing chronic pain. She was able to resume her normal activities three months post-surgery. She was followed up for the next six months to ensure no post-operative complications, no recurrence of pain and patient is satisfied with her outcome.

## Discussion

Peroneal disorder is an under-diagnosed cause of lateral ankle pain where the commonest among peroneal pathology is isolated peroneus brevis tear (70%) followed by mixed peroneal tear (21%) and isolated peroneus longus tear (9%)^4^. Clinical findings can be vague and the result from MRI at times are not accurate, as suggested from our case of an isolated peroneus longus tear being reported as mixed peroneal tendinopathy.

An isolated peroneal tendon tear is commonly associated with acute ankle inversion injuries^3^. Long term tendon and ligament injuries can lead to ankle instability which later can cause ankle osteoarthritis. However, those with peroneal injuries presenting with insidious worsening of pain occurs in chronic conditions such as peroneal tendon subluxation, lateral ankle instability and anatomical variation within the retromalleolar groove that may lead to tear as a result of microtrauma or tendon weakening such as in patients receiving steroid injection for pain or patients with diabetes mellitus^2, 3^. Peroneus longus tear frequently occur in regions of high shear stress, more commonly at the cuboid tunnel, peroneal tubercle and also at the tip of the lateral malleolus^2, 3^. Peroneal brevis tear is usually found within the retromalleolar sulcus and the tear is associated with high mechanical stress within the area.

Patients with peroneal tendinopathy can be initially started with trial of conservative therapy. This includes NSAIDs, activity modification, physical therapy and even a period of immobilisation especially if there is tenderness or swelling^3^. Physical therapy with stretching and strengthening activities helps alleviate the symptoms. Other modality of therapy including massage, ultrasound and electrical stimulation has also been advocated to alleviate symptom^3^. However, symptoms are likely to persist in most of the patients especially in chronic long-standing lateral ankle pain in which patients with non-resolving symptoms will be offered surgery^1, 4^.

Intra-operative findings may differ from MRI reading. During exposure, extra care is taken in protecting the sural nerve. Resection is performed in cases of low-lying peroneal muscle belly or if there is presence of fraying. Most of the chronic cases, degenerative tear is present end this can be treated with tendon debridement and tubularization of the tendon^1, 4^. If the tear more than 50% of the tendon, a tenodesis between the peroneus longus and peroneus brevis can be performed^1, 2^. It is important to localise the site of the anastomosis in order to avoid impingement and stenosis which can cause further friction and non-resolving of the pain. Post-operative immobilisation is important at earlier post-operative stage prior to starting physical therapy^1, 2^.

In summary, patients with a peroneal tendon tear, who have pain or decreased function and have failed conservative management, may benefit from operative management. Patients who have peroneal tears with minimal to moderate degeneration can have excellent pain relief, improvement of overall function and return to full activity after debridement and primary repair with tubularization with optimum post-operative care and follow-up.
